# Bright Light Therapy for MDD in Children and Adolescents: a narrative review of literature

**DOI:** 10.1192/j.eurpsy.2022.1418

**Published:** 2022-09-01

**Authors:** R. Vadukapuram, C. Trivedi, Z. Mansuri, K. Shah, A. Reddy

**Affiliations:** 1 Icahn School of Medicine at Mount Sinai, Psychiatry, New York, United States of America; 2 Texas Tech University Health Science Center at Permian Basin, Psychiatry, Midland, United States of America; 3 Boston Childrens Hospital, Psychiatry, MA, United States of America; 4 Griffin Memorial Hospital, Psychiatry, Norman, United States of America; 5 Virginia Tech Carilion School of Medicine, Psychiatry, Roanoke, United States of America

**Keywords:** MDD, Bright light therapy

## Abstract

**Introduction:**

Major Depressive Disorder (MDD) is a common mood disorder diagnosed in children and adolescents. Bright light therapy has been effective for seasonal affective disorders, however its role in the treatment of MDD is under studied.

**Objectives:**

Our objective is to evaluate if bright light therapy (BLT) is a practical approach in treating Child and Adolescents having MDD.

**Methods:**

We performed an extensive literature search using a wide range of MeSH terms in PubMed, PubMed Central and Google Scholar. We reviewed the literature for studies (published between 1983-2021) assessing the efficacy of BLT in the treatment of MDD in children and adolescents.

**Results:**

The final search results yielded 8 randomized clinical trials and 1 case report from 1983 to 2021. BLT showed a superior effect in children and adolescents with MDD compared to the control group in the majority of the randomized trials and a case report. In six studies BLT showed good effect, however in a study by Magnusson et al. and Sonis et al., found a milder degree of improvement in depression symptoms when compared to the control group. In the majority of the studies, patients’ age range was 7 years 18 and in most of the studies, patients were not on antidepressants.

**Conclusions:**

The use of BLT in children and adolescents suffering from MDD can be a promising alternative method of biological treatment, which is effective as well as well tolerated. Future long-term studies on large sample size are necessary in this field.

**Disclosure:**

No significant relationships.

